# A solution to prevent secondary flow in adherent cell cultures

**DOI:** 10.1242/bio.045294

**Published:** 2019-07-15

**Authors:** Peter Szaraz, Matthew Librach, Poonam Mander, Banafshe Hoseini, Max Librach, Farwah Iqbal, Clifford Librach

**Affiliations:** 1 Create Program Inc., Suite 412, Toronto, Ontario M5G 1N8, Canada; 2 Dept. Physiology, University of Toronto, Toronto, Ontario M5S 1A8, Canada; 3 Dept. of Obstetrics and Gynecology, University of Toronto, Toronto, Ontario M5G 1E2, Canada

**Keywords:** Adherent cell culture, Cell differentiation, Mesenchymal stem cells, Feeder cell culture

## Abstract

High quality cell cultures require reliable laboratory practices. Today's small-scale *in vitro* cell culture format is dominated by circular topology vessels, with the inherent disadvantage of secondary flow induced each time the cell cultures are repositioned. The secondary flow generates uneven sedimentation and adherence that negatively impacts cell culture quality. Here we show a modification of the circular culture vessel that abrogates these disturbances. Cell culture wells were augmented with a central column to diminish secondary flow. Human carcinoma cell lines (BeWo, JEG-3), mesenchymal stem cells [human umbilical cord perivascular cells (HUCPVC)] and mouse embryonic fibroblasts (MEF) were cultured in both column-augmented and regular culture wells. Human carcinoma cell cultures showed even cell densities and significantly more viable cells in column-augmented vessels. In FTM HUCPVC cultures, cell surface MSC marker (CD90, CD105) expression and cell differentiation-related gene expression patterns were significantly more homogeneous in column-augmented vessels. MEF cells in column-augmented culture vessels showed a more consistent expression of IGF-1. Column-augmented cell culture vessels significantly improve the homogeneity of adherent cell cultures by mitigating the adverse effect of the secondary flow.

This article has an associated First Person interview with the first author of the paper.

## Abstract (precis)

**Summary:** Secondary flow in small scale cultures can crucially affect cell phenotype and function. A simple modification of the classical round-shaped cell culture vessels can minimize the adverse effect of secondary flow.

## INTRODUCTION

### Principles of small-scale adherent cell cultures

Small-scale cell culture experiments most commonly aim to characterize the identity and potential of adherent eukaryotic cells. Whether the *in vitro* culture is based on primary tissue isolates or established cell lines, pathological malformations or therapeutic cells for pre-clinical testing, the first stage of assessment is essentially always the same setup: a transparent plastic vessel providing a growth surface for the cells and a liquid phase of cell culture. The principles of small-scale cultures date back to Julius Richard Petri's research in the 1880s (Petri, 1887). Petri's microbiological practices introduced the cylindrical cell culture vessel that has been in use in various iterations ever since. Eukaryotic cell research adopted this cell culture vessel topology and various sizes of the cylindrical cell culture dishes are utilized worldwide.

Reliability and reproducibility require sufficient biological and technical replicates in every experimental study, to comply with guidelines of good laboratory practice (GLP). In any academic or industrial establishment, maintenance and investigation of mammalian cell cultures begins with the traditional low-volume plasticware, typically made of polystyrene (PS) or polyethylene (PE). The most commonly used are 10 cm and 6 cm diameter culture dishes. Six- to 96-well plates provide a sufficient surface for 103-106 adherent cells to develop, ideally as monolayer cultures. Cell densities in these cultures are set to suit the purpose of the culture and typically range between 20–70% surface coverage, and this is referred to as ‘confluency’. Procedures and assessments requiring limited cell–cell connections and high relative cell surface (such as transfection protocols) or identifiable cell margins (microscopy) favor lower cell confluency, while tests conducted on larger cell populations (flow cytometry and immunoblotting) seek higher yields from a single vessel. Regardless of preferred confluence, consistent growth of the culture is key to ensure any treatments to the cell population are evenly applied and the subsequent results sufficiently represent the entirety of the cells in the culture.

In addition to the practical aspects of the analysis, the experimental design must consider that the cell density in a culture fundamentally impacts the micromilieu and as such, the biology of the cells. Higher local cell densities increase autocrine and paracrine effects (Jayatilaka et al., 2017), and cell–cell connections can affect cell proliferation (Ribatti, 2017) and cell fate (Graffmann et al., 2018). When cell density reaches a critical level, monolayer cultures often develop three-dimensional structures with multiple layers of cells. Once cells have multiple neighbors and lack direct exposure to culture media, the limited availability of nutrients and oxygen invariably changes cellular phenotype and function. High local cell densities can lead to a sub-optimal and eventually uninhabitable environment, and ultimately necrosis or programmed cell death.

In order to have sufficient control over cell cultures and guarantee stability and reproducibility, physical parameters are pre-set and monitored constantly. These parameters include temperature, oxygen and carbon dioxide levels, and, if possible, the culture media composition. The control over the culture's cell density is just as crucial because cells are essential environmental factors for each other. Setting defined cell numbers at seeding and performing counting at the time of harvest accounts for the overall cell numbers per culture unit. However, local alterations in cell confluency and cell growth can lead to inhomogeneities in the cell culture and result in variability within the cultivated cell population. Uneven seeding, adhesion and proliferation can lead to these heterogeneities that, depending on the cell type, can give rise to uncontrolled, phenotypically diverse sub-populations. Cell culture protocols are stringent practices used in an attempt to homogenize cell populations and eliminate factors that might jeopardize even growth and homogeneity.

### The tea-leaf paradox and secondary flow

**Fig. 1.**
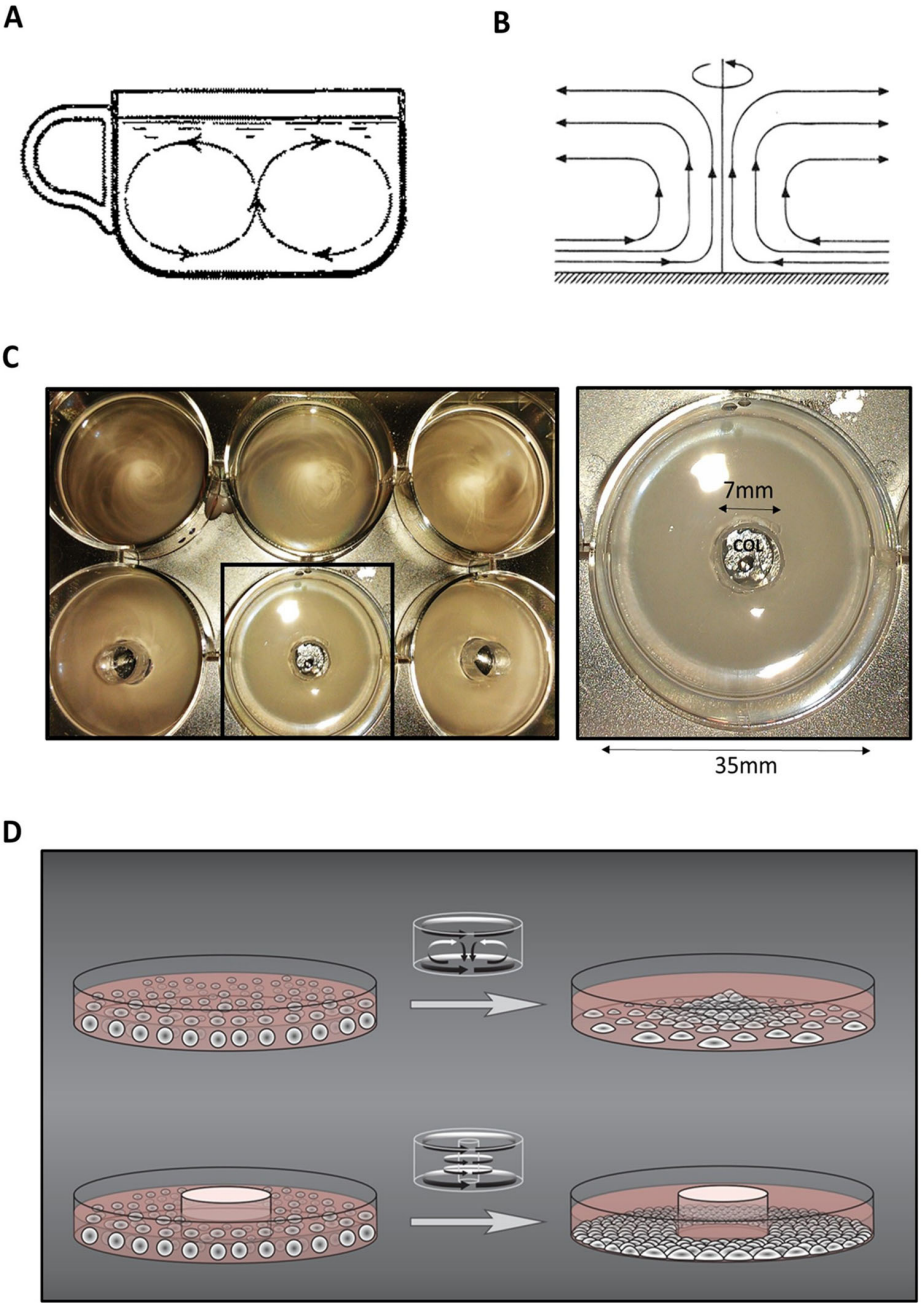
**The secondary flow affects the sedimentation of suspended particles in regular but not in column-augmented vessels.** (A) Secondary flow in stirred tea cup (modified from A. Einstein, 1926). (B) Secondary flow trajectories moving along the bottom towards the axis of the circular vessel in a centripetal manner. (C) Sedimentation of dispersed particles in regular and column-augmented wells of a six-well culture plate (see Movie 1). Box: measurements of culture well and column diameter. (D) Schematic depiction of presumed sedimentation and growth of cell suspensions in regular and column-augmented culture vessels.

The so called ‘tea-leaf paradox’ was known for centuries and posed a non-trivial fluid mechanics puzzle until 1926, when Albert Einstein published his article on the matter (Yogananda and Albert Einstein, 2000; Einstein, 1926). Tea leaves on the bottom of the stirred cup gather in the center rather than migrating to the edges of the cup (Fig. 1A) because sediment in a curved fluid system descends and collects along the smaller curve and lifts along the longer curve (Fig. 1B). The phenomenon is explained through Baer’s Law (Yogananda and Albert Einstein, 2000; Einstein, 1926) and is analogous to the formation of a river's banks by how its water meanders. Einstein introduced the definition and description of secondary flow in fluid dynamics as the direct driving force of these events.

Secondary flow occurs in any fluid phase moving along a curved path. It is most prominent in cylindrical vessels where fluids are subject to forces that set the liquid in circular motion. Dispersed particles in the liquid move with the secondary flow and sediment along the smaller curve or the center of a cylindrical vessel.

### Secondary flow in adherent cell cultures

Intuitively, similar liquid dynamics would apply in the case of conventional cylindrical cell culture vessels. Any movement of the culture dish, that is, any force introduced along the horizontal plain, induces primary and secondary flow in the fluid phase, which in this case is the cell culture media. If floating cells are present in the fluid phase, secondary flow inevitably leads to uneven sedimentation on the bottom of the dish (Fig. 1D). In extreme cases this can result in an over-confluent central region and a sparsely covered, low confluency peripheral region of the dish (Fig. 1D). Uneven adherence and growth in cylindrical cell culture vessels is broadly recognized and has been accepted as an inevitable inconvenience of traditional small-scale cultures (Research Gate). Although other effects of the fluid flow on cells such as shear stress (Salek et al., 2012) soluble ligand transfer (Batsilas et al., 2003) were investigated, the population-level alterations of the cell culture due to uneven sedimentation and growth are not properly addressed.

When translating basic research into large-scale cell production, for purposes such regenerative medicine cell therapy, it is imperative that cell identity and function are preserved as originally optimized in small-scale cultures. A common feature of large-scale, bioreactor-based, cell cultures is the pursuit of even parameters throughout the reactor volume, in order to produce a homogeneous cell product. This is achieved by the sufficient influx of nutrients and soluble gases in a convection or flow-through driven liquid phase and, in the case of adherent cells, by providing a suitable growth surface where cells can expand without contact inhibition. Overall, bioreactors provide homogenous cell cultures that are necessary to gain a reliable and consistent cell product.

We hypothesized that: (1) secondary flow is induced when handling cylindrical culture vessels, leading to uneven attachment and growth of adherent cells; and (2) placing a circular object in the center of the culture vessel prior to seeding will decrease secondary flow and increase homogeneity in an adherent cell culture.

### Mesenchymal stromal cells

Mesenchymal stromal cells or mesenchymal stem cells (MSC) are receiving increased attention and attracting funding in the field of regenerative medicine. Since the introduction of the concept of adult stem cells (Caplan, 1991) and the evidence of their presence in virtually every tissue of the human body (Caplan, 2015), many regenerative attributes have been discovered and described. In addition to their ability to differentiate into mesenchymal lineages, their paracrine properties are also of interest and are an important consideration for medical applications (Murphy et al., 2013). A cell type's ability to adhere and proliferate on plastic surfaces is a requirement of its potential as a therapeutic candidate (Dominici et al., 2006) and is the case of MSC. Notably, cells with increased differentiation potential and paracrine properties, such as adherent MSC cultures, are especially sensitive to cell density.

We are investigating a novel young source of MSC isolated from the perivascular region of the human umbilical cord (Hong et al., 2013). These pericyte-like cells called human umbilical cord perivascular cells (HUCPVC) can be isolated from term and first trimester (FTM) umbilical cord tissue. We have demonstrated that both cell types have high proliferation rates and express a set of paracrine factors, contributing to their promise as candidates for cell therapy applications. FTM HUCPVC have been proven to have a differentiation potential that outperforms older sources of MSC (Szaraz et al., 2017). To maintain culture control, it is imperative that small-scale adherent cultures maintain homogenous confluency within and throughout each plate or well prior to each assay or procedure.

## RESULTS

We tested our hypothesis by utilizing six-well tissue culture plates (35 mm diameter wells) and straight, cylindrical polystyrene inserts attached to the center of flat bottom wells with biologically inert silicon grease. Assigned wells were augmented with 7 mm diameter central attachments, henceforth referred to as ‘columns’. Consequently, the ratio of the inner and outer radii of the attachment surface was 1:5 in column-augmented wells (Fig. 1C). The column insert decreased the growth surface by less than 5%.

For rapid visualization of liquid flow and sedimentation in regular and column-augmented wells, latex beads (4 µm) were transferred into each well of the six-well plates as a 1:5 dilution with PBS (3 ml). Beads were noted to visibly begin to sediment after the tissue culture plate was randomly agitated on the horizontal plane (Fig. 1C; Movie 1). In regular, unaltered wells sedimentation inhomogeneities were observed instantly with a high density of beads appearing in the wells' central area (Fig. 1C, upper row; Movie 1). However, in column-augmented wells (Fig. 1D, bottom row; Movie 1) latex beads showed even sedimentation and distribution throughout the wells.

**Fig. 2.**
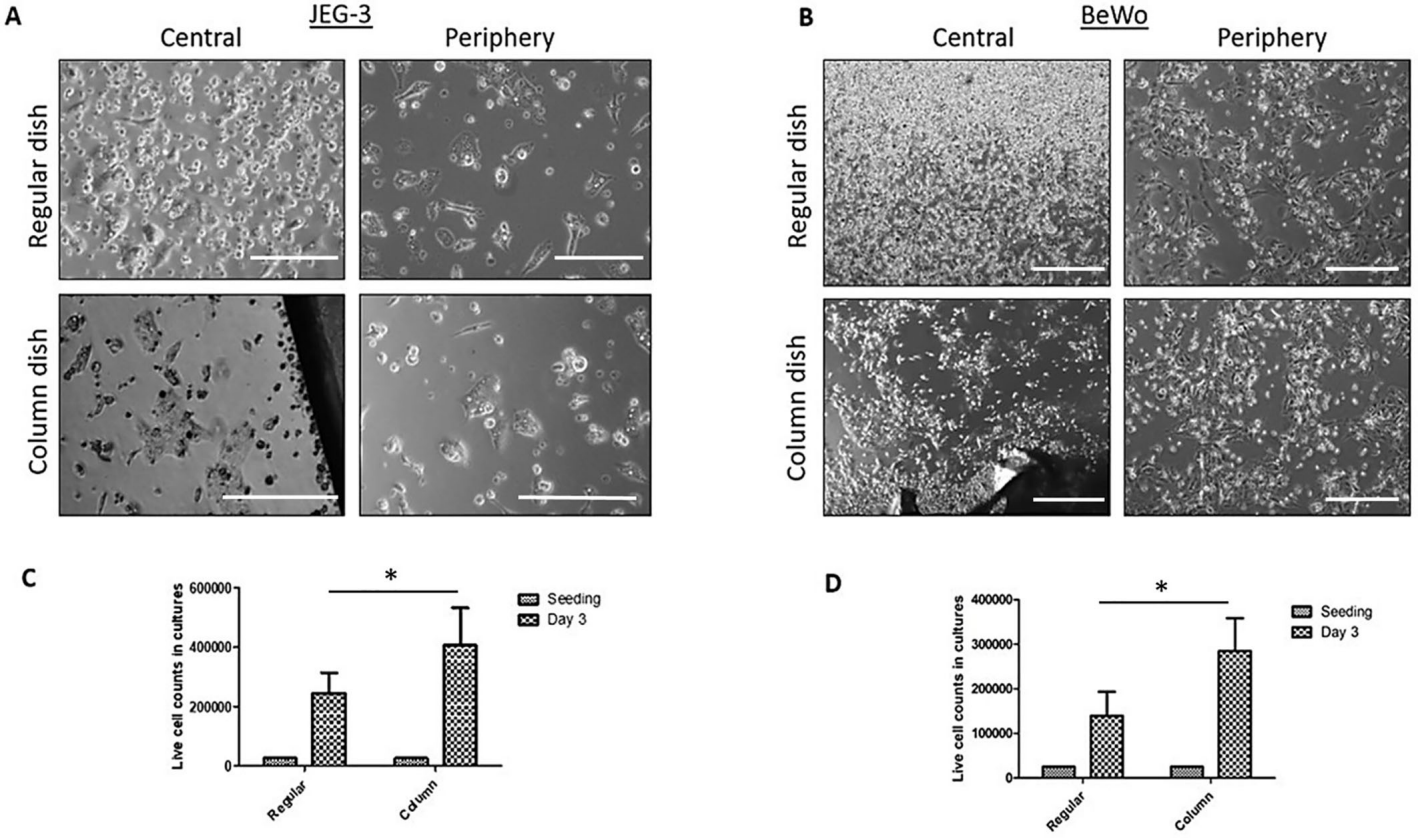
**Cell growth and viable cell counts in JEG-3 and BeWo cultures are diminished in regular culture vessels compared to column-augmented vessels.** (A) Phase contrast microscopy-JEG-3 cells in central and peripheral areas of regular and column-augmented culture dishes (six-well plate) 3 days after cell seeding (2.5×10^4^ cells per well). (B) Phase contrast microscopy-BeWo cells in central and peripheral areas of regular and column-augmented culture dishes (six-well plate) 3 days after cell seeding (2.5×10^4^ cells per well). (C,D) Quantification of live cell counts both JEG-3 (C) and BeWo (D) cultures show significantly higher cell counts in column-augmented culture wells compared to regular ones (*P<0.01, *n*=5). Scale bar: 400 µm.

**Fig. 3.**
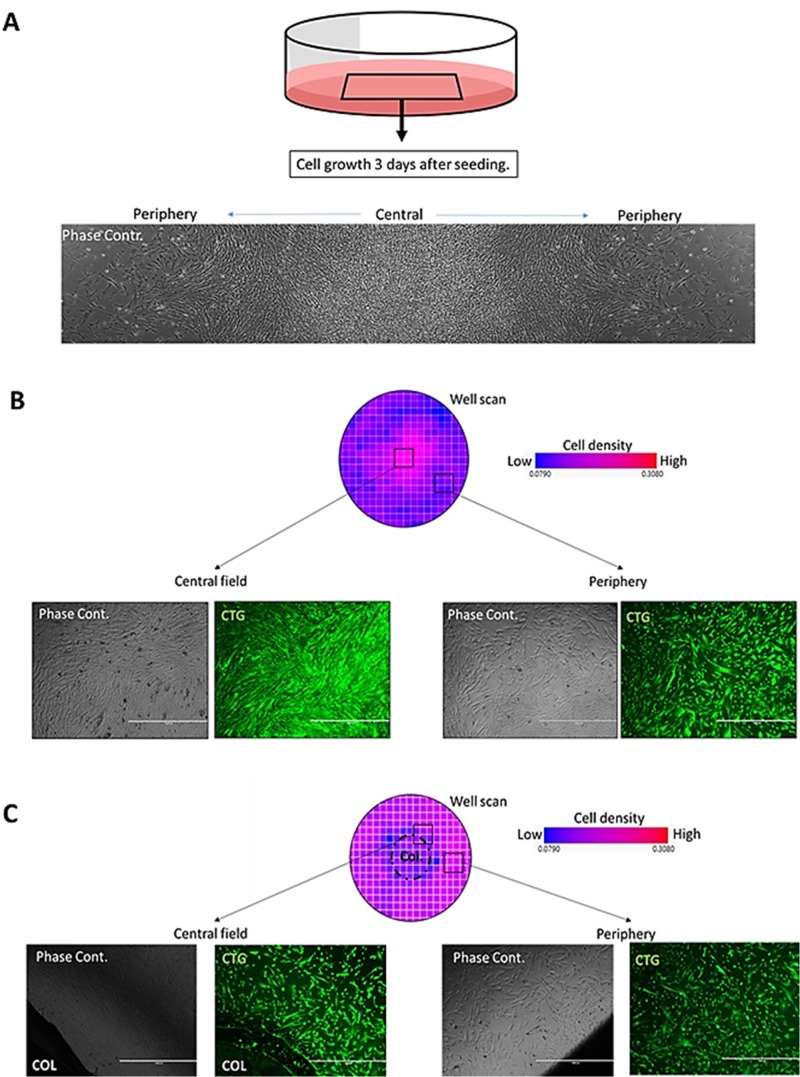
**Cell growth-altering effect of secondary flow in regular, but not in column-augmented FTM HUCPVC cultures.** (A) Growth surface coverage of FTM HUCPVCs in six-well culture 3 days after cell seeding (2.5×10^4^ cells in 2 ml media). Composed phase contrast microscopy image shows cell density gradient established from the central to the peripheral region. (B) Live fluorophore (CellTrackerGreen™) staining of regular FTM HUCPVC culture at 3 days after cell seeding. Fluorescence well scan and fluorescence microscopy confirm high central growth density as sparse peripheral cell growth. (C) Fluorescence well scan and fluorescence microscopy show even cell growth in column-augmented cell cultures. Wells of the same six-well plate were acquired and analyzed. Scale bars: 400 µm.

Immortalized cell lines from both human and mammalian animal sources are a convenient tool for cell biology evaluations. While these cell types are generally believed to provide robust and replicable cultures, it is reported that growth densities can affect the outcome of assessments due to paracrine (Jayatilaka et al., 2017) and cell contact-related effects (Ribatti, 2017). For our experiments two choriocarcinoma cell lines (JEG-3 and BeWo) were expanded in regular and column-augmented culture wells for cell growth and viability assessment. For both cell types and vessel types, 2.5×10^3^ cells were seeded in each well of six-well culture plates in alpha-MEM (10% FBS) and cultured for 3 days. At the time of seeding, cell culture plates were subjected to the movements occurring in our usual laboratory practices, with no additional shaking or agitation applied to the culture plates. 3 days after seeding, cultures were imaged by phase contrast microscopy (Fig. 2A,B) and cells were lifted for live cell counting (Fig. 2C,D). Both JEG-3 and BeWo cultures in regular culture wells showed high cell densities and a high number of floating cells in the central region compared to the peripheral regions of their respective culture wells. Column-augmented cultures did not present with such differences. When comparing live cell counts of JEG-3 and BeWo cultures respectively (day 3), a significantly higher number of viable cells were found in the column-augmented wells compared to regular culture wells for both cell types (Fig. 2C,D). FTM HUCPVC were pre-loaded with viable CellTracker™ Green (2.5 µg/ml, 30 min) and seeded on six-well plates with or without a central column. 10^5^ cells were transferred as single cell suspensions into each well in 3 ml of culture media (alpha-MEM, 10% FBS, 1% sodium pyruvate). In each six-well plate three wells were unaltered and three were column-augmented. With all wells physically connected, any force applied on the plate affected all wells identically. To mimic the manipulation of the culture plate and the conventional mixing practices that aim to distribute cells evenly in culture plates, we moved the plate 3–4 times rapidly along the horizontal plane before placing it into the incubator. After 48 h of incubation (37°C, 5% CO_2_) phase contrast and fluorescence microscopy were performed to assess cell growth and confluency (Fig. 3). Bright field microscopy composed images (Fig. 3A) showed a gradient of cell confluency that increased from the edge of the well and reaching a maximum in the central area of the well. Fluorescence microscopy confirmed major differences in cell confluency based on location in the regular culture wells (Fig. 3B). Fluorescence well scans were performed on the adherent cell cultures to quantitatively assess cell densities, confirming up to ten times higher density in the central region of a regular well compared to the peripheral areas (Fig. 3B). In contrast, both microscopy and quantitative fluorescence well scans showed that the presence of column inserts in the wells of a six-well plate resulted in virtually homogenous cell growth (Fig. 3C).

**Fig. 4.**
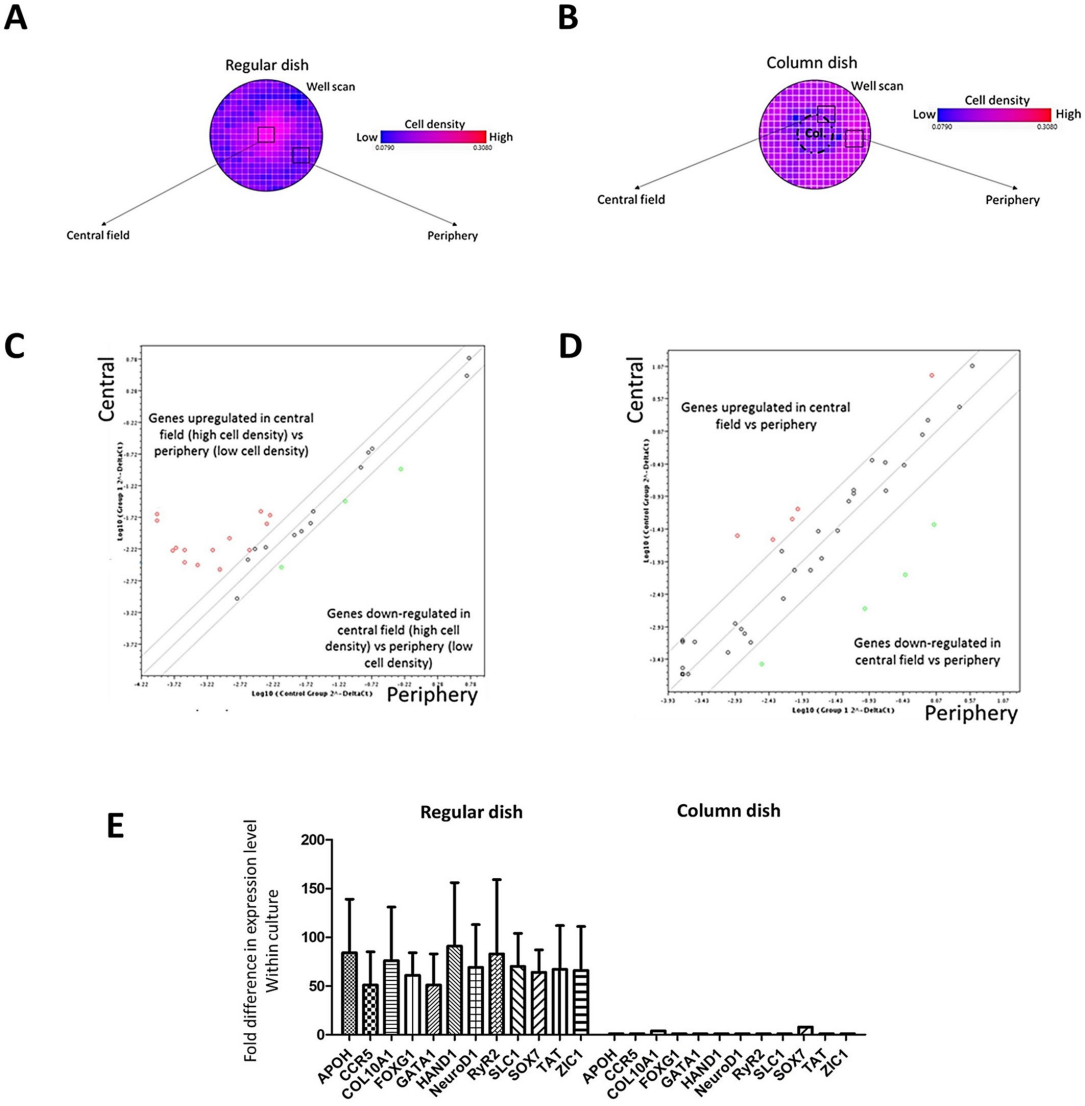
**Lineage commitment and stem cell differentiation-related gene expression levels are altered in regular and but not in column-augmented FTM HUCPVC cultures.** (A,B) Sampling map of regular culture well defining central and peripheral regions for comparative analysis. (C) Gene expression level ratios in cells isolated from central versus peripheral regions of regular cell culture well. (D) Gene expression level ratios in cells isolated from central versus peripheral regions of regular cell culture well. Values expressed as log10 (2^normalizedΔct). (E) Lineage commitment gene expression level differences in cell cultures depending on growth location. Values expressed as fold difference, central versus peripheral fields. Regular culture dishes presented with higher differences of gene expression within the same cultures compared to column-augmented wells. *N*=4.

Adherent cells were extracted from central and peripheral areas of regular and column-augmented six-well cultures of FTM HUCPVCs and processed for stem cell differentiation quantitative PCR array (Qiagen) (Fig. 4). Samples obtained from regular culture wells showed major differences in the expression of multiple genes represented in the array (Fig. 4C,D) depending on the sampling location within the well. Gene expression quantification indicated more than 50-fold higher expression of cell fate and lineage commitment-related genes: HAND1, FABP7, CD3E, APOH, FOXG1, NANOG, HNF4A, NEUROD 1RYR2 and TAT (Fig. 4E). This result suggests that gene expression and epigenetic state of initially undifferentiated MSC change based on local cell density in the culture (Fig. 4A). Cells isolated from different regions of column-augmented dishes did not show these alterations in gene expression levels (Fig. 4B).

MSC are regularly detected and characterized by specific cell surface markers that can individually or in combination identify MSC populations, distinguishing them from other cell types and indicate their undifferentiated state (Dominici et al., 2006). CD105 (Endoglin) and CD90 (THY1) are both routinely used to identify adult stem cells from various tissue sources (Lange et al., 2005; Feng-Juan et al., 2014). While CD105 and CD90 cell surface expression is often classified as simply marking positive and negative cell populations. Both markers are under specific and continuous expression control that is related to the extracellular environment.

**Fig. 5.**
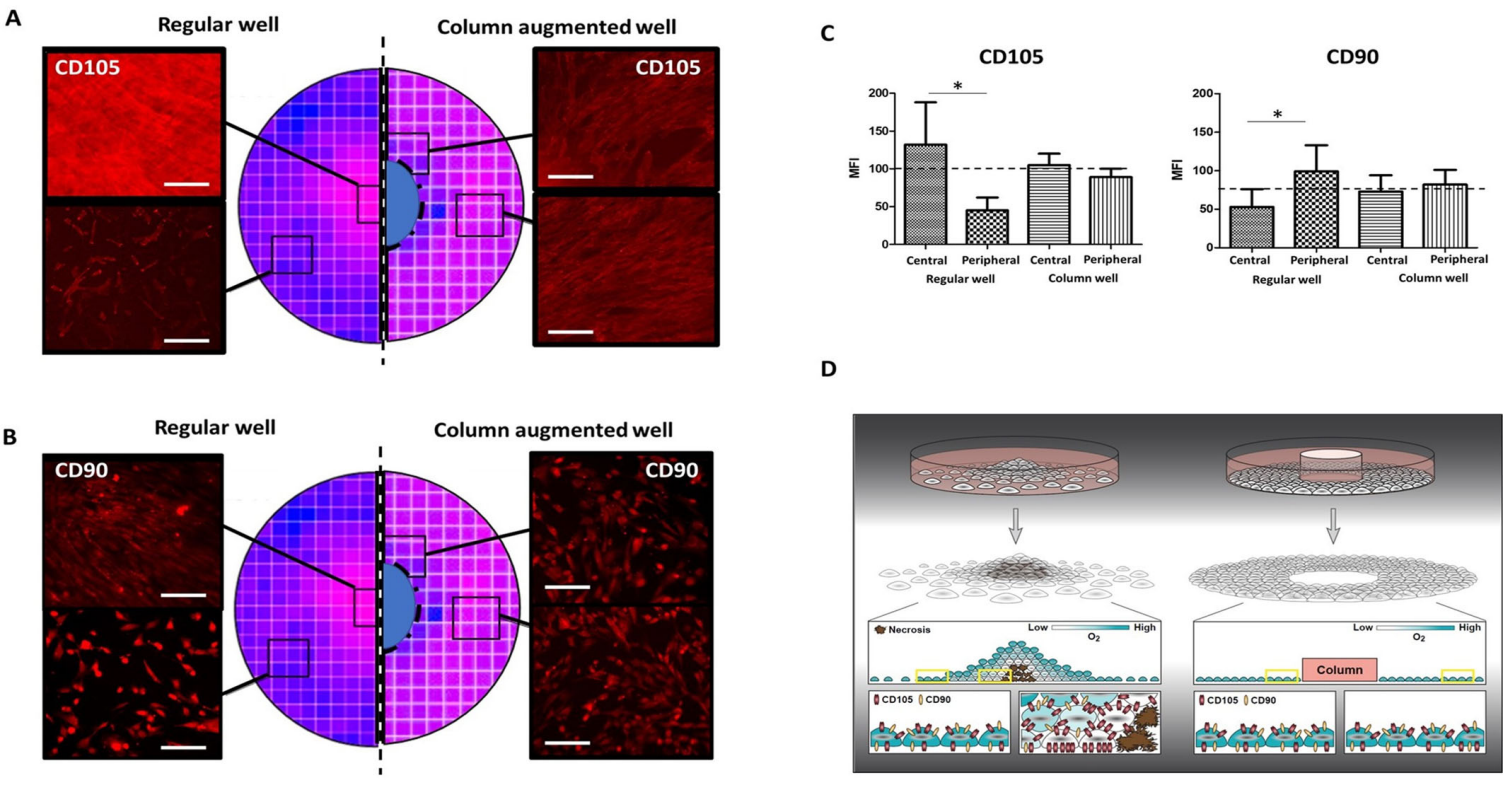
**CD105 and CD90 cell surface expression of FTM HUCPVC is altered in regular but not in column-augmented culture wells.** (A) CD105 live immunostaining in peripheral and central regions of regular and column-augmented wells. (B) CD90 live immunostaining in peripheral and central regions of regular and column-augmented wells. (C) Quantification of CD105 and CD90 expression levels by flow cytometry analysis shows significant (*P<0.03, *n*=4) differences between central and peripheral regions of the regular well cultures, but not in column-augmented ones. (D) Theoretical schematics of cell growth density effect on CD105 and CD90 expression in regular and column-augmented cultures. Scale bars: 200 μm.

Challenging the paradigm of the constitutive representation of MSC-markers, research shows that CD105 expression can be controlled by oxygen availability in the cellular micromilieu (Sánches-Elsner et al., 2002; Mark et al., 2013), while CD90 representation on the cell surface can depend on the extent of cell–cell connections (Kim et al., 2017). In both cases, these markers have a negative correlation, respectively, with their above-mentioned parameters. We aimed to investigate whether these parameters are altered locally in the regular cell culture well to the level where cell surface markers used to describe MSC show detectable differences between cells growing in the center or the periphery of the well. FTM HUCPVC cultures in both regular and column-augmented wells were cultured for 48 h after seeding. At this time point, column-augmented wells contained cell monolayers exclusively, while regular plates had over confluent central surface areas (Fig. 5A). Cell culture media was removed and fluorophore-conjugated primary antibodies for the native forms of MSC surface markers CD105 and CD90 were added to each well. Anti-CD105 (PE) and anti-CD90 (APC) were applied individually in 1:40 dilution (PBS, FBS 5%) and incubated for 20 min with the adherent cells. After washing the wells with PBS (5 min), fluorescence microscopy was performed to detect immunostaining (Fig. 5A,B). Regular culture wells that displayed major differences in cell density (depending on the distance from the central area of the growth surface) showed different staining intensities for each antibody. While CD105-related fluorescence signal intensity was higher in the central areas and lower in the sparsely confluent peripheral regions in the regular culture wells, the opposite trend was observed for CD90 staining. Column-augmented cultures had virtually no difference in fluorescence signal for either CD105 or CD90 when comparing central or peripheral fields (Fig. 5B).

Flow cytometry analysis was performed on FTM HUCPVC cell suspensions retrieved from regular and column-augmented culture wells to quantify cell surface expression of CD105 and CD90 individually. Central and peripheral growth areas were lifted from both regular and column-augmented culture wells separately and analyzed as individual samples (Fig. 5C). Single cell suspensions of 5×10^4^ cells were incubated with either CD105 (PE) or CD90 (APC) antibodies and analyzed using MACSQuant digital flow cytometry. Mean fluorescence intensities (MFI) were exported and graphed to compare signals contributed to cells grown in the central or peripheral regions of each culture well (Fig. 5C). The quantification agreed with the previous observations and showed significant differences in both CD105 and CD90 signals when comparing cells from the central and peripheral fields of the regular culture wells. While CD105 expression was significantly higher and CD90 expression was significantly lower in cells grown in the high confluency central areas of the regular culture wells compared to the low confluency peripheral areas, cells from column-augmented dishes displayed signals in both cases that were between the extremes observed in the regular dish (Fig. 5C, scattered line). In addition to the observation that higher cell growth homogeneity leads to increased phenotypical homogeneity, these results indicate that cell surface marker expression is a continuum that is a function of parameters in the cellular micro-environment. These results are consistent with literature reports on how CD105 and CD90 expression can be altered by cell growth and cellular micromilieu (Fig. 5D), highlighting the importance of homogenous and controlled cultures when cultivating and characterizing MSC.

**Fig. 6.**
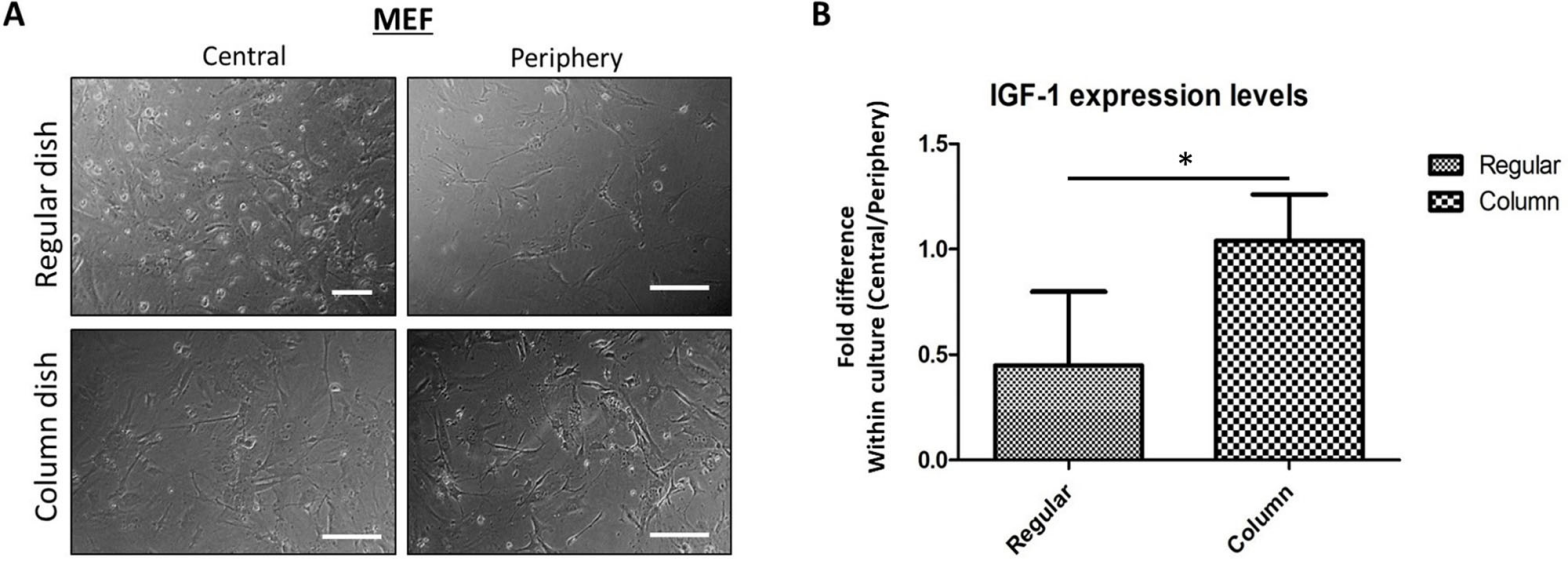
**Cell growth area-dependent differences detected in IGF-1 expression of MEF cells in regular but not in column-augmented cultures.** (A) MEF cells grown in regular and column-augmented six-well culture plates for 3 days (2.5×10^4^ cells in 2 ml media). (B) IGF-1 expression levels in central and peripheral growth regions of culture vessels were analyzed by qPCR and expressed as fold difference within culture. Regular culture dishes presented with significantly altered expression levels compared to column-augmented cultures. (*P<0.03, *n*=4). Scale bars: 200 μm.

Feeder layer cell cultures are regularly applied for the maintenance and propagation of stem cells *in vitro*. A high-quality feeder culture is required to provide a stable, homogenous niche to support stem cells and preserve their undifferentiated state. IGF-1 is a growth factor that is essential for the self-renewal of embryonic (ES), pluripotent (PS) and neural (NS) stem cells and whose expression level has become a quality control measure when comparing feeder cells (Wang et al., 2007). Mouse embryonic fibroblasts (MEF) are one of the most generally applied cell types for establishing feeder layer cultures, with IGF-1 expression being a crucial feature in their ability to generate the favorable stem cell niche. For our experiments, MEF cells were seeded in regular and column-augmented culture vessels at 5×10^4^ per cm^2^ cell density and cultured for 3 days (alpha-MEM, 10% FBS). Cell culture plates were handled according to standard laboratory practices and included plate transfer for bright field microscopy from the biosafety cabinet to a humidified incubator. No additional agitation was applied to the plates. After 3 days, MEF cultures from regular wells showed similar differences in cell densities that we observed for the cell types discussed previously (Fig. 6A): while central regions of the regular culture wells presented with a higher cell density, the column-augmentation successfully abrogated this. MEF cells were lysed (RLT) in a location-dependent manner, separating cells from the central and peripheral regions of each culture dish, and processed for qPCR analysis. IGF-1 expression levels were normalized to β-actin and expressed as a ratio of central and peripheral fields (Fig. 6B). While MEF cells from regular culture wells showed an average 50% decrease in IGF-1 expression when comparing central growth fields to peripheral ones, no such difference was found in MEF cultures grown in column-augmented cultures (Fig. 6B).

## DISCUSSION

Cell biology is a rapidly developing area of biomedical research; however, the basic principles of small-scale adherent cell cultures have remained fundamentally conserved since Petri’s *in vitro* methods were established (Petri, 1887). The cylindrical culture vessel is still commonly used in cell biology laboratories worldwide. The work presented here demonstrates that fluid mechanics, in particular the induction of secondary flow, in the small-scale, circular culture vessels can significantly impact a cell culture's quality.

Cultures of immortalized cell lines are common tools for basic science and research given their general robustness and ease of handling. However, we found that inhomogeneities emerging in BeWo and JEG-3 cultures cultivated in circular vessels negatively impact their growth and viability. Highly proliferative cells suitable for culturing and *in vitro* evaluations may also originate from healthy sources. Stem cells of embryonic, adult and, more recently, extra-embryonic sources, are currently in the spotlight of regenerative medical applications. To gain reliable information on cellular phenotype and function so that findings and practices can be translatable to high-scale cell production and therapeutic applications, stem cell cultures need to be maintained and handled with high fidelity. Using a young, extra-embryonic source of MSC (FTM HUCPVC) we demonstrated that inhomogeneities in the growth of these adherent cell cultures can occur during routine growth in conventional, cylindrical culture vessels. Finally, we tested MEF to assess whether their function of generating supportive niche for stem cells is impacted by the inhomogeneities in the typical culture system. Our experimental results were consistent for each cell type tested: the differences in confluency found concurrently in the same culture vessel can lead to alterations in the cells' immunophenotype and gene expression regulation and can ultimately lead to the emergence of phenotypically different sub-populations. Therefore, gaining control over the homogeneity of cell cultures ensures the reliability of the evaluation and increases the fidelity of the transition towards large-scale applications.

We have demonstrated here, with a simple modification, that augmenting standard circular culture vessels with a central insert, or ‘column’, growth homogeneity of the adherent culture can be significantly increased, and the adverse effects of uneven cell growth can be virtually eliminated. Based on these findings, we suggest that such an approach should be strongly considered when working with adherent cell cultures.

## MATERIALS AND METHODS

Six-well cell culture plates were purchased from Corning. Sterile polypropylene inserts of 35 mm in diameter were applied as columns attached to the bottom surface of selected culture wells using sterile silicon grease (Eppendorf).

4 µm silicone beads were used for cell-free sedimentation assessment (Thermo Fisher Scientific). Silicone beads were washed with PBS and transferred into culture wells in a 1:5 dilution in 3 ml of PBS. Sedimentation was visualized after rapid agitation.

JEG-3 and BeWo human choriocarcinoma cell lines were ordered from ATCC and cultured in alpha MEM (Gibco) supplemented with 10% fetal bovine serum (FBS, Hyclone) and 1% Penicillin-Streptomycin (Gibco). For cell growth and viability assessments, 2.5×10^4^ cells were seeded in 2 ml media per well in six-well cell culture plates. Cells were analyzed 3 days after seeding.

Three lines of FTM HUCPVC (passage 4–6) were cultured in alpha MEM (Gibco) supplemented with 10% fetal bovine serum (FBS, Hyclone) and 1% Penicillin-Streptomycin (Gibco). FTM HUCPVC were transferred into culture wells as single cell suspensions of 5×10^4^ cells in 3 ml of media (as described above). Rapid horizontal agitation was applied to culture plates containing both regular and column-augmented wells. Cell cultures were incubated for 48 h at 37°C, 5% CO_2_, in humidified incubators. Bright field (Zeiss) and fluorescence microscopy (EVOS, Life Technologies) images were acquired after 48 h of incubation. Images were taken of central and peripheral fields of both regular and column-augmented wells.

Cell Tracker Green (Invitrogen) life fluorescent dye was applied to adherent cell cultures at 2.5 µg/ml for 30 min to load cells for fluorescence microscopy and well-scan. Quantitative fluorescence well scans were performed on fluorophore-labelled cell cultures using a digital plate reader (FilterMax F5, Molecular Devices). Immunofluorescence labelling of FTM HUCPVC was performed using primary conjugated antibodies: anti- CD105 PE (RnD) and anti-CD90 APC (BD). Antibodies were applied in a 1:40 dilution in PBS supplemented with 3% FBS (Hyclone) for 30 min. Fluorescence imaging of immuno-labelled cells was performed with an EVOS digital microscope (Life Technologies). For flow cytometry analysis single cell suspensions of 10^5^ cells were prepared and incubated with primary conjugated antibodies (1:40 dilution in PBS with 3% FBS). Digital flow cytometry was performed using MACSQuant (Miltenyi). Data analysis was performed with FlowJo software. Statistical analysis was performed using ANOVA.

RNA was isolated from 10^5^ cells per sample for qPCR analysis. Cells were lysed and RNA was purified using the RNEasy Plus Mini Kit according to the manufacturer's instructions. Quantitative PCR was performed using the Human Cell Lineage Identification PCR Array (PAHS-508R, Qiagen) run in a Corbett RotorGene qPCR machine (Qiagen). Gene expression levels were normalized to integrated housekeeping controls (HPRT, GAPDH, ACTB). Results were analyzed and exported by the RT2 Profiler PCR Array Data Analysis Platform (SABiosciences, Qiagen). Results were expressed as a fold-change between samples gathered from the peripheral and central areas of the culture wells.

Mouse Embryonic Fibroblast Feeder cells (Applied Stem Cell Inc.) were cultured in DMEM (Sigma Life Science) supplemented with 10% FBS (Hyclone), 1% Penicillin-Streptomycin (Gibco), and 1 mM Sodium Pyruvate (Gibco). Cell cultures were maintained at 37°C, 5% CO_2_ in a humidified incubator. After 72 h in culture, cells were lysed from the central and peripheral growth surfaces separately using RLT buffer (Qiagen). RNA was purified using the RNeasy Mini Kit (Qiagen). cDNA was synthesized with First Strand RT2 (Qiagen) and qPCR was performed using Quant Studio5 (Thermo Fisher Scientific) using SYBR Green Master Mix (Qiagen). Gene expression levels were normalized to housekeeping control β-actin; primers were synthesized by ACGT Corp. (Toronto). Mouse IGF-1 Forward: AAAGCAGC-CCCGCTCTATCC, Reverse: CTTCTGAGTCTTGGGCATGTCA, Mouse β-actin Forward: GGCACCACACCTTCTACAATG, Reverse: GGGGTGTTGAAGGTCTCAAAC. Results were analyzed with the RT2 Profiler PCR Array Data Analysis Platform (SABiosciences, Qiagen). The thermal profile for qPCR was 95°C for 10 min followed by 40 cycles of 15 s at 95°C, with 2 min at 65°C. Relative expression of the target genes was calculated with the 2¯ΔΔct method.

## Supplementary Material

Supplementary information
