# Supplementary material for: A solution to prevent secondary flow in adherent cell cultures
Source: Biol Open. 2019 Jul 15;8(7):bio045294. doi: 10.1242/bio.045294 (PMC6679401; doi:10.1242/bio.045294)
Supplement: Supplementary information [file biolopen-8-045294-s1.pdf]

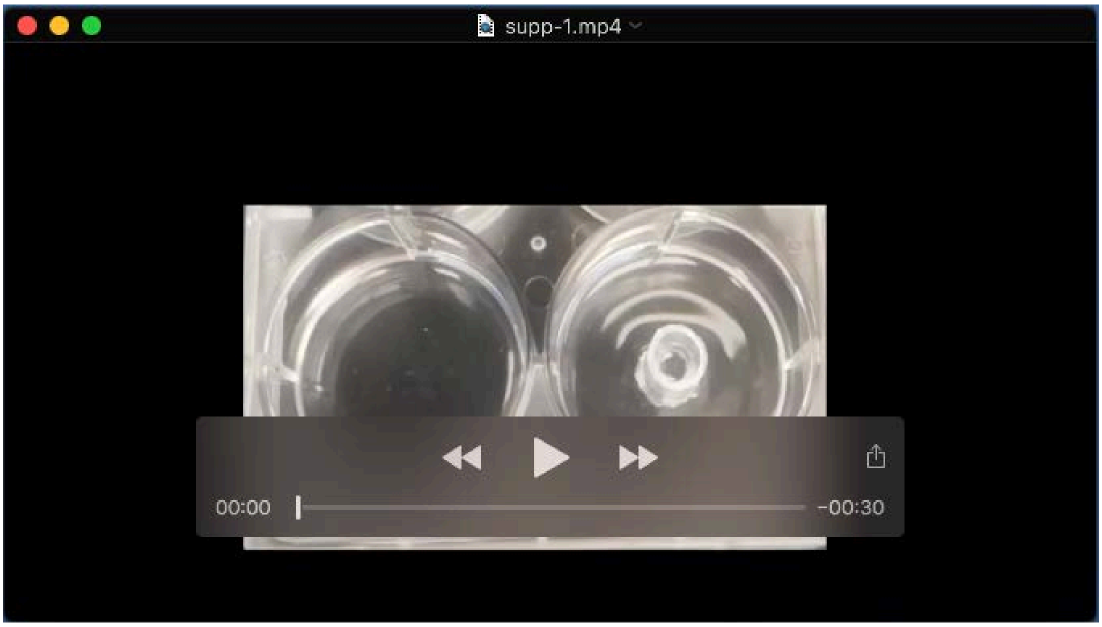

Movie 1. Particle sedimentation after agitation in regular and column-augmented wells of 6-well tissue culture plate. Silicone beads (ThermoFisher) of 4 µm diameter were suspended in 3 ml PBS in a 1:5 volumetric dilution and transferred into regular and column-augmented wells. Tissue culture plate then was placed on horizontal shaker platform and agitated at 250 rpm for 3 seconds. While beads visibly collected to the central region of the regular well as an result of fluid movement, an even flow and sedimentation was observed in column-augmented wells.
